# Medroxyprogesterone Acetate Impairs Amyloid Beta Degradation in a Matrix Metalloproteinase-9 Dependent Manner

**DOI:** 10.3389/fnagi.2020.00092

**Published:** 2020-04-07

**Authors:** Keyana N. Porter, Saumyendra N. Sarkar, Duaa A. Dakhlallah, Mya E. Vannoy, Dominic D. Quintana, James W. Simpkins

**Affiliations:** ^1^Department of Pharmaceutical and Pharmacological Sciences, West Virginia University School of Pharmacy, Morgantown, WV, United States; ^2^Department of Physiology and Pharmacology, West Virginia University School of Medicine, Morgantown, WV, United States; ^3^Department of Microbiology, Immunology and Cell Biology, West Virginia University, Morgantown, WV, United States; ^4^Department of Neuroscience, Center for Basic and Translational Stroke Research, Rockefeller Neuroscience Institute, West Virginia University, Morgantown, WV, United States

**Keywords:** matrix metalloproteinases, medroxyprogesterone acetate, amyloid-beta (Aβ), amyloid-beta (Aβ) degradation, Alzheimer’s disease, hormone therapy, glucocorticoid receptor

## Abstract

Despite the extensive use of hormonal methods as either contraception or menopausal hormone therapy (HT), there is very little known about the potential effects of these compounds on the cellular processes of the brain. Medroxyprogesterone Acetate (MPA) is a progestogen used globally in the hormonal contraceptive, Depo Provera, by women in their reproductive prime and is a major compound found in HT formulations used by menopausal women. MPA promotes changes in the circulating levels of matrix metalloproteinases (MMPs), such as MMP-9, in the endometrium, yet limited literature studying the effects of MPA on neurons and astroglia cells has been conducted. Additionally, the dysregulation of MMPs has been implicated in the pathology of Alzheimer’s disease (AD), where inhibiting the secretion of MMP-9 from astroglia reduces the proteolytic degradation of amyloid-beta. Thus, we hypothesize that exposure to MPA disrupts proteolytic degradation of amyloid-beta through the downregulation of MMP-9 expression and subsequent secretion. To assess the effect of progestins on MMP-9 and amyloid-beta, *in vitro*, C6 rat glial cells were exposed to MPA for 48 h and then the enzymatic, secretory, and amyloid-beta degrading capacity of MMP-9 was assessed from the conditioned culture medium. We found that MPA treatment inhibited transcription of MMP-9, which resulted in a subsequent decrease in the production and secretion of MMP-9 protein, in part through the glucocorticoid receptor. Additionally, we investigated the consequences of amyloid beta-degrading activity and found that MPA treatment decreased proteolytic degradation of amyloid-beta. Our results suggest MPA suppresses amyloid-beta degradation in an MMP-9-dependent manner, *in vitro*, and potentially compromises the clearance of amyloid-beta *in vivo*.

## Introduction

Alzheimer’s disease (AD) is a neurodegenerative disease with three key pathological hallmarks: the progressive accumulation of extracellular deposits (amyloid plaques); aggregates of intracellular protein (neurofibrillary tangles); and loss of neurons and synapses (reviewed by Haass and Selkoe, 2007). Extracellular amyloid plaques are mostly composed of amyloid beta-peptide (Aβ), which is generated by proteolytic cleavage of amyloid precursor protein (APP; De Strooper, 2010). APP is recognized by alpha-secretase (α;-secretase), which cleaves the precursor protein, promoting the non-amyloidogenic cleavage of APP, which has been shown to promote neuroprotection and memory enhancement (Ghiso and Frangione, 2002). However, as reviewed by Chen (2015); with aging, α-secretase becomes progressively inefficient. This causes APP to be truncated by non-specific proteases such as β-secretase and γ-secretase, promoting the amyloidogenic cleavage of APP (Holsinger et al., 2002; Yang et al., 2003; Zhang et al., 2014; Carroll and Li, 2016). Cleavage of APP at the β- and γ-secretase sites produces the 40 or 42 amino acid fragments of Aβ (Aβ_1–40_ and Aβ_1–42_) and subsequently, Aβ peptide is released into the extracellular space (Chen, 2015; Xu et al., 2016). Extracellular Aβ assumes several conformational states ranging from monomers to soluble oligomers and fibrils. These polymers of Aβ quickly aggregate and form the amyloid plaques which are characteristic of the disease (Pryor et al., 2012).

The clearance of Aβ is mediated by several Aβ-degrading zinc-metalloproteinases, including endothelin-converting enzyme (ECE), angiotensin-converting enzyme (ACE), insulin-degrading enzyme (IDE), neprilysin (NEP) and matrix metalloproteinases (MMPs; Saido and Leissring, 2012). MMPs are a family of highly regulated, zinc-dependent enzymes that are produced by neurons and astroglia (Dzwonek et al., 2004). These enzymes are initially secreted as catalytically inactive pro-enzymes (proMMP-9), which are bound to endogenous inhibitors, named tissue inhibitors of metalloproteinases (TIMPs), through the C-terminal domain of each molecule. A disturbance of this complex (proMMP-9\textbullet TIMP), by a proMMP activator (MMP-3), causes the pro-enzyme (proMMP-9) to dissociate, which becomes fully activated, and proceeds with the degradation of its substrates (Ogata et al., 1995; Rosenblum et al., 2007). Functionally, MMPs are extremely diverse, with regulatory roles in many important cellular processes including synaptic plasticity and cognition, neuroinflammation, blood-brain barrier (BBB) integrity, cell migration, survival, and apoptosis, as reviewed by Vafadari et al. (2016). MMPs are overexpressed during various pathological conditions such as stroke, epilepsy, schizophrenia, and neurodegeneration (Vafadari et al., 2016). Their expression can be induced by a large variety of factors, including cytokines, growth factors, metal ions, antibiotics, and hormones (Van den Steen et al., 2002; Vandooren et al., 2017). In the context of AD, Aβ exposure induces the expression and secretion of MMPs from cultured neurons, reactive astroglia, and neuroblastoma cells (Deb and Gottschall, 1996; Deb et al., 2003; Talamagas et al., 2007; Mizoguchi et al., 2009). When secreted from astroglia, proMMP-9 is immediately cleaved into an active form (MMP-9) in the extracellular compartment, thus contributing to the maintenance of the balance between Aβ production and clearance (Ogata et al., 1995; Akiyama et al., 2000; Wegiel et al., 2000). In contrast to other Aβ-degrading proteases, MMP-9 is known to degrade Aβ fibrils *in vitro*, Aβ plaques *in situ*, and Aβ *in vivo* (Backstrom et al., 1996; Yan et al., 2006; Yin et al., 2006; Hernandez-Guillamon et al., 2015). Consequently, modulation of MMP-9 levels can therefore impact the clearance of Aβ and promote its deposition.

Medroxyprogesterone Acetate (MPA) is a widely used, synthetic progestin that is primarily found in the contraceptive, Depo Provera^®^, and HT, Prempro^®^ and Premphase^®^. Merlo and Sortino (2012) utilized estrogen to induce the Aβ-degrading activity of MMP-9, establishing MMP-2 and MMP-9’s contribution to the neuroprotective effect of the hormone *in vitro* (Merlo and Sortino, 2012). Our work focused on MPA, and its ability to modulate the levels of MMP-9, and by extension the degradation of Aβ. MPA exerts adverse effects on cognition, substantiating the risk of dementia in menopausal women (Shumaker et al., 2003, 2004), promoting memory impairments in menopausal animal models (Braden et al., 2010, 2011, 2017), and inducing amnesia in case study reports of premenopausal women (Gabriel and Fahim, 2005). MMP-9 is critical for cellular processes involved in learning and memory, as it regulates dendritic spine morphology, maintains late-phase long term potentiation (LTP), and controls postnatal brain development (Nagy et al., 2006; Michaluk et al., 2011; Kamat et al., 2014; Gorkiewicz et al., 2015; Reinhard et al., 2015; Kaczmarek, 2016). MPA has also been shown to alter MMP-9 activity and production in BV2 microglial, cancer, and epithelial cells (Di Nezza et al., 2003; Hwang-Levine et al., 2011; Allen et al., 2019). Additionally, the secretion of MMP-9 was found to be negatively impacted by MPA in the aforementioned cell types (Deb and Gottschall, 1996; Deb et al., 2003; Hwang-Levine et al., 2011; Allen et al., 2019). It is currently unknown if hormonal modulation of glial-secreted MMP-9, using MPA, impacts degradation of Aβ. The pharmacological inhibition of MMP-2 and MMP-9, using both specific and non-specific enzymatic inhibitors, has also been shown to attenuate astroglia cell-mediated Aβ degradation (Yin et al., 2006). Therefore, MPA-mediated reduction of glial-secreted of MMP-9 would likely result in dysregulated APP processing, fostering conditions that would disrupt clearance of Aβ. On the premises that: (1) this commonly used progestin negatively impacts the secretion of this Aβ-degrading enzyme, in the aforementioned cell types; and (2) inhibition of glial secretion of MMP-9 reduces the proteolytic degradation of amyloid-beta, our central hypothesis is that MPA diminishes the secretion of MMP-9, thereby reducing the degradation of amyloid-beta.

Here, we provide evidence that illustrates the connection of MPA, MMP-9, and Aβ. We found that MPA influences Aβ degradation by modulating the expression and/or activity of Aβ-degrading enzymes in a glial cell line (C6). C6 rat glial cells are a commonly used glial cell line and have also been identified as a useful cell line to study hormone action in glia (Kumar et al., 1986; Buchanan et al., 2000; Su et al., 2012b). We demonstrate that MPA significantly reduces enzymatic activity and secretion of MMP-9 and that MPA significantly reduces the degradation of Aβ. Together, these data implicate MPA in a negative effect on the Aβ-degrading enzyme, MMP-9. These data support a potential role where MPA perturbs Aβ clearance mechanisms, indicating the necessity for *in vivo* investigations of MPA’s influence on AD-related pathology.

## Materials and Methods

### Cell Culture

The C6 rat glial cell line was obtained from the American Type Culture Collection (ATCC #CCL 107). C6 cells were maintained in Hyclone Dulbecco’s modified Eagle’s medium (DMEM)/high glucose (Thermo Fisher Scientific, Waltham, MA, USA) supplemented with 10% fetal bovine serum (FBS; Atlanta Biologicals) and 1% penicillin/streptomycin (Thermo Fisher Scientific, Waltham, MA, USA). Once cells reached 80% confluency, they were trypsinized and spun down at 325.5 × g for 3 min. Cells were counted with a Nexcelom Bioscience Cellometer AutoT4 (Lawrence) and seeded at a density of 1 × 10^6^ cell/well in 6-well cluster plates, incubated overnight at 37°C in 5% CO_2_.

### Treatments

Serum-supplemented media was removed and the cell monolayer was washed once with phosphate buffer saline (1× PBS) and fresh-serum free media (OptiMEM; Thermo Fisher Scientific, Waltham, MA, USA), containing either 0.1% dimethyl sulfoxide (DMSO; Millipore Sigma), various concentrations of Medroxyprogesterone Acetate (MPA; 10 nM, 100 nM, 1 μM, and 10 μM; Millipore Sigma), GM6001 (1 μM; Calbiochem, La Jolla, CA, USA), or RU486 (2 μM; Millipore Sigma). All treatments were diluted in serum-free medium to a final DMSO concentration of 0.1%. Cell viability was always above 90% as assessed by Calcein AM (Thermo Fisher Scientific, Waltham, MA, USA). C6 cells were incubated at 37°C in 5% CO_2_ for 48-h or 72-h. The conditioned media was collected and used to assess MMP-9 enzymatic activity (by gelatinase zymography), quantify extracellular MMP-9 (by ELISA), and assess proteolytic degradation of amyloid-beta (by Western blot). Supernatants were stored at −80°C and thawed on ice during experimental analyses. Repeated freeze-thaw cycles were avoided by aliquoting the samples. The cells were collected and used to quantify intracellular MMP-9 protein levels (by ELISA) and assess MMP-9 mRNA expression (by qRT-PCR).

### Cell Viability

Cell viability was assessed using Calcein AM (Life Technologies) and reconstituted at 2 mM in dimethylsulfoxide. About 50,000 C6 cells were seeded in a black-walled clear bottom 96 well plate (Corning; #3601). C6 cells were treated with 10 nM–10 μM of MPA. After exposure to MPA (10 nM–10 μM) for 72-h, the plate was washed three times with PBS 1×. A total of 100 μl of 1 μM Calcein AM was added to the wells. The plate was incubated at 22°C in the dark for 30 min. The plate was read using a BioTek Synergy H1 Hybrid reader (Winooski, VT, USA).

### Lactate Dehydrogenase Assay

Cell death after MPA treatment was assessed using the Pierce^™^ lactate dehydrogenase (LDH) Cytotoxicity Assay (Thermo Fisher Scientific, Waltham, MA, USA) kit. Reaction substrates were prepared as per the manufacturer’s instructions. LDH assay was performed with the media from the black-walled clear bottom 96 well plate. Forty-five minutes before the end of the 72-h exposure period, 10 μl 10× lysis buffer was added to one control well, and the plate was placed back in the incubator. After the exposure period, 50 μl of media was carefully removed from each well and transferred to a new 96 well clear-bottom assay plate. Next, 50 μl of the LDH reaction mixture was added to each well, and the plate was incubated for 30 min at room temperature, protected from light. The reaction was stopped by adding 50 μl of LDH stop solution to each sample. The plate was read using the BioTek Synergy H1 Hybrid Reader (BioTek) at an absorbance of 490 nm and 680 nm.

### Assay of MMP-9 Activity by Gelatin Zymography

The gelatinolytic/proteolytic activity of MMP-9 secreted into the culture medium was determined with gelatin zymography by electrophoresis of serum-free conditioned media (CM) collected from confluent C6 cells, following (Frankowski et al., 2012). Briefly, CM freed of cell debris by centrifugation were mixed with Laemmli sample buffer (Bio-Rad; #1610747) lacking reducing agents. Electrophoresis in precast Novex polyacrylamide zymogram gels (Invitrogen) was performed at a constant voltage of 125V for 90 min in SDS Tris-Glycine Buffer, followed by a series of three 15 min washes in 1× renaturation buffer (Invitrogen). The gels were transferred to 1× Developing buffer (Life Technologies) for 30 min with gentle shaking and then placed at 37°C for and 18 h incubation. The following day, the gels were stained in a solution with 45% ethanol, 54% sterile diH_2_O, 1% acetic acid, and 0.125 g Coomassie brilliant blue R-250 (Sigma) between 45 min to 1-h. The gels were then transferred to de-staining solution #1, containing 25% ethanol, 10% acetic acid, and 65% diH_2_O for 45 min. De-staining solution #1 was decanted and then replaced by a de-staining solution #2, containing 5% ethanol, 7.5% acetic acid, and 87.5% diH_2_O for 1–3 h. Areas of gelatinolytic degradation appeared as transparent bands on the blue background. Gels were imaged using ChemiDoc^TM^ XRS+ System (Bio-Rad, Hercules, CA, USA). Images were acquired using Bio-Rad Quantity One^TM^ software. The Novex Sharp Pre-stained Protein Standard (Invitrogen) was used to identify MMP species or the MMP-9 Active, Human, Recombinant (Millipore Sigma; #PF024-5UG) was used as a reference standard, showing MMP-9 gelatinolytic activity at 67 kDa. The bands in the gel are quantified using ImageJ 1.38X(NIH).

### Intracellular and Extracellular MMP-9 Levels

CM samples and cell lysates were analyzed with a matrix metalloproteinase-9 (MMP-9) ELISA kit following the product manuals (R&D Systems Quantikine^™^; #RMP900). The results were calculated from the standard calibration curves on internal standards. After adding stop solution, optical density (OD) was measured at 450-nm with correction wavelength at 550-nm immediately using BioTek Synergy H1 Hybrid plate reader (BioTek). The final readings were obtained by subtracting 450-nm from 550-nm OD reading to correct for optical imperfections of the microplate reader. A standard curve was generated with reagents provided in the kit and the sample values were read against the standard to determine MMP-9 concentrations in each treatment.

### RNA Isolation and Quantitative Real-Time PCR

Cells were seeded at 1 × 10^6^ cells/well in a 6-well plate and left to reach ~80% confluency throughout 24-h. The media was gently aspirated from cells and replaced by 2 ml of OptiMEM Reduced Serum Media (Thermo Fisher Scientific, Waltham, MA, USA), supplemented with 1% penicillin/streptomycin (Thermo Fisher Scientific, Waltham, MA, USA), or the various concentrations of MPA. After a 12-h exposure period, cells were collected using a Cell Lifter (Corning, Corning, NY, USA), washed with 1× PBS, and pelleted by centrifugation at 325.5 × g for 3 min, followed by the addition of 1 ml QIAzol Lysis Reagent for RNA purification (Qiagen).

Total RNA was purified by using the miRNeasy Mini Kit (Qiagen) as per the manufacturer’s instructions. For all lysate samples, 200 μl chloroform was added, and vortexed for 1 min followed by incubation on ice for 5–10 min. Next, the samples were centrifuged for 15 min at 12,000× *g* at 4°C. The upper aqueous phase (~300 μl) was transferred to a new collection tube and mixed with 600 μl 100% ethanol. The solution was then placed on an RNeasy MinElute spin column and centrifuged at 8,000 × g for 15 s. The flow-through was discarded and 700 μl buffer RW1 (20% Ethanol, 900 mM guanidinium isothiocyanate (GITC), 10 mM Tris-HCl pH 7.5) was added to the spin column and centrifuged at 8,000× *g* for 15 s. Two washes of 500 μl buffer RPE (80% Ethanol, 100 mM NaCl, 10 mM Tris-HCl pH 7.5) were then performed, with the first lasting 15 s and the second lasting 2 min. The RNeasy MinElute spin column was then placed in a new collection tube and spun at 8,000 × g for 5 min to dry the column membrane. Lastly, the column was placed in another collection tube and 40 μl RNase-free water was added to the center of the membrane and incubated for 1 min, and then centrifuged at 8,000 × *g* for 1 min ending with the purified RNA eluted in the collection tube.

RNA concentrations for each sample were measured using Nanodrop 2000 spectrophotometer (Thermo Scientific). For cDNA synthesis, 0.75 μg total RNA was reverse transcribed using the miScript II RT kit (Qiagen). A reaction mix (20 μl total volume) was made using 4 μl 5× miScript HiFlexBuffer, 2 μl 10× miScript Nucleics Mix, 2 μl miScript Reverse Transcriptase Mix, and 12 μl Template RNA/nuclease-free water. Before use for quantitative real-time PCR (qRT-PCR), cDNA was diluted in nuclease-free water, at a ratio of 1:10.

Expression of MMP-9 mRNA was determined using target-specific RT^2^ primer assays and the RT^2^ SYBR^®^ Green PCR kit (Qiagen; 5 μl SYBR^®^ Green; 0.5 μl Target Primer; 4.5 μl diluted cDNA). qRT-PCR reactions were performed in duplicate for each sample, using the CFX384 Touch^TM^ RT PCR Detection System (Bio-Rad) for 45 cycles as follows: 15 s at 94°C, 30 s at 55°C, 30 s at 70°C. Negative control reactions were included as wells containing only master mix and nuclease-free water (no template cDNA). The expression levels of target genes in cell lysates was standardized against Adenylyl cyclase-associated protein 1 (CAP-1; IDT). Quantification of PCR amplified mRNA specific cDNA was done by the comparative cycle threshold CT method (2^−ΔΔCT^). Ct values of mRNA were subtracted from the average Ct of the internal controls, and the resulting ΔCT was used in the equation: relative copy numbers = (2^−ΔΔCT^).

### Amyloid-Beta Preparation and Amyloid-Beta Degradation in Astroglia-Conditioned Medium

Synthetic Aβ_1–42_ (Invitrogen; #30112) was prepared from lyophilized Aβ_1–42_ monomers that were suspended in 167 μl of HPLC grade water (Thermo Fisher Scientific, Waltham, MA, USA) and incubated at room temperature for 5 min. The dissolved Aβ_1–42_ was then diluted to 230 μM by adding 833 μl of Ca^2+^-free phosphate-buffered saline (PBS) and incubated for 48-h at 37°C for polymerization. After polymerization, synthetic Aβ_1–42_ (230 μM) at a final concentration of 23 μM was freshly prepared and added to either serum-free media (SFM), untreated CM, MPA-treated CM, or GM6001-treated CM of C6 glial cells. The mixtures were then incubated at 37°C (Yin et al., 2006). After 24 h, samples were collected and residual Aβ_1–42_ was analyzed by Tris-Glycine—Western blotting.

Western blot was performed using denaturing 4–20% Novex Wedgewell Tris-Glycine SDS gels (Invitrogen) with 90 min electrophoresis at 125V, 30 mA and iBlot Gel Transfer Stacks (Invitrogen) with 7 min of electrical blotting. The polyvinylidene difluoride (PVDF) membrane (Invitrogen) with proteins transferred was blocked by Odyssey Blocking Buffer (LI-COR Biosciences; #927-40000) for 1-h and was incubated with primary 6E10 (1:1,000, Biolegend; #803016) overnight and then with secondary (anti-mouse conjugated with fluorescence; LI-COR) at room temperature for 90 min. Bands were visualized using LI-COR Odyssey IR Imager (LI-COR). Quantification using densitometric analysis was performed using Odyssey imaging systems (LI-COR Biosystems, Lincoln, NE, USA). Densitometry signal for each range of oligomers [low molecular weight aggregates (<15 kDa), intermediate-sized oligomers (~15–55 kDa), high molecular weight oligomers (>56 kDa), or the entire lane for total Aβ_1–42_] was normalized relative to the signal of the control lane (SFM) and fold change over untreated cells was plotted.

### Statistical Analyses

All biological experiments were repeated at least three times with *n* = 3–16 plates/wells per treatment. The results from the experiments are reported as means ± SEM. All quantitative data were assessed for significance using a one-way ANOVA with Dunnett’s *post hoc* test. All results were analyzed by GraphPad Prism 8.0 software (GraphPad Software). A *p*-value < 0.05 was used to establish significance.

## Results

### Medroxyprogesterone Acetate Reduces MMP-9 Enzymatic Activity

**Figure 1:**
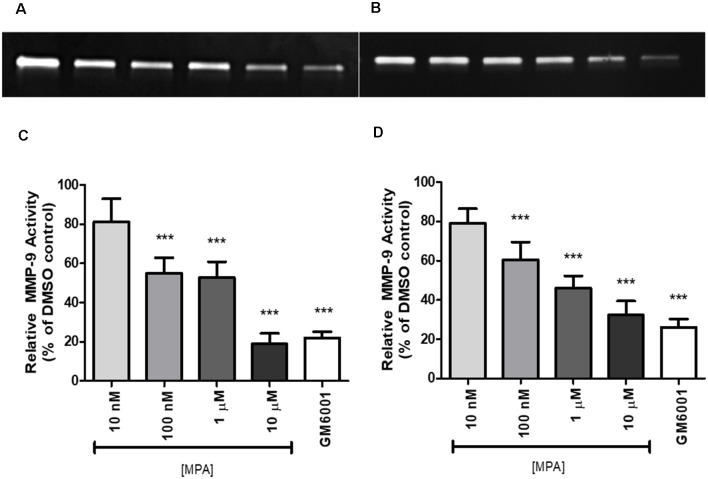
Medroxyprogesterone Acetate (MPA) reduced the gelatinolytic activity of MMP-9 in C6 glial cells. **(A,B)** Representative zymograms showing MMP-9 activity of MPA-treated conditioned media (CM) determined by gelatin zymography. After a 48-h (left) or 72-h (right) incubation period in serum-free media, supernatants obtained from 1 × 10^6^ cells were analyzed by gelatin zymography. DMSO at 0.1% was used as the vehicle control and constitutively showed MMP-9 gelatinolytic activity (lane 1). Upon MPA treatment, MMP-9 gelatinolytic activity was significantly decreased (lanes 2–5). GM6001 (lane 6) was used as a negative control for active MMP-9. **(C,D)** Densitometric analysis of conditioned media from astroglia determined by gelatin zymography. C6 glial cells were incubated for 48-h (left) or 72-h (right) with increasing concentrations of MPA and GM6001, a non-specific MMP inhibitor. Results are expressed as percentage of activity of treated to untreated cells (mean ± SEM). MMP-9 gelatinolytic activity of untreated cells is expressed as 100%. MPA significantly decreased MMP-9 enzymatic activity in a dose-dependent manner (*P* < 0.001) compared with that found in untreated cells. The results are representative of five independent experiments. ***p < 0.001.

To study the effect of Medroxyprogesterone Acetate (MPA) on matrix metalloproteinase-9 (MMP-9) activity, we incubated C6 cells for 48 and 72-h with increasing concentrations of MPA (10 nM—10 μM). The conditioned media (CM) were then collected and analyzed by gelatin zymography. This technique allows for the visualization of both active and proenzyme (inactive) forms of gelatinases (MMP-2 and MMP-9; Frankowski et al., 2012). Untreated C6 cells show constitutive expression of active MMP-9 denoted by the 92 kDa gelatinase band (Figures 1A,B; Supplementary Figures S3, S4), with no detections of bands representative of MMP-2 activity. Densitometric analysis of zymograms obtained in five different experiments indicated that 48-h incubation of C6 cells with MPA inhibited MMP-9 activity in a dose-dependent manner, with significant reductions occurring at the three highest concentrations (100 nM, 1 μM, and 10 μM) compared to untreated control cells (Figures 1C,D). As expected, GM6001, an MMP inhibitor, suppressed MMP-9 enzymatic activity by 80%. This dose-dependent, inhibitory effect of both MPA and GM6001 on enzymatic activity persisted at 72-h.

To determine whether the inhibitory effect of MPA on MMP-9 activity was due to interference with the activation process of MMP-9, post-secretion, we incubated C6 cells with medium alone. After 48-h, the CM was collected, divided into aliquots, and treated with increasing concentrations of MPA. The media was then incubated for 24 more hours at 37°C and analyzed by gelatin gel zymography. The inhibitory effect of MPA was lost, suggesting that MPA does not directly interact with MMP-9 and confirming the interference with the activity of the protease is cell-mediated (data not shown).

To exclude the possibility of drug toxicity as the reason of its inhibitory effect, C6 cells were incubated with increasing concentrations of MPA (10 nM, 100 nM, 1 μM, and 10 μM) or 1 μM GM6001, then cell viability using Calcein AM and LDH release were assessed. MPA did not cause any appreciable cellular toxicity, even at the highest concentration used (10 μM; Supplementary Figure S1).

### Medroxyprogesterone Acetate Reduces MMP-9 Production

**Figure 2:**
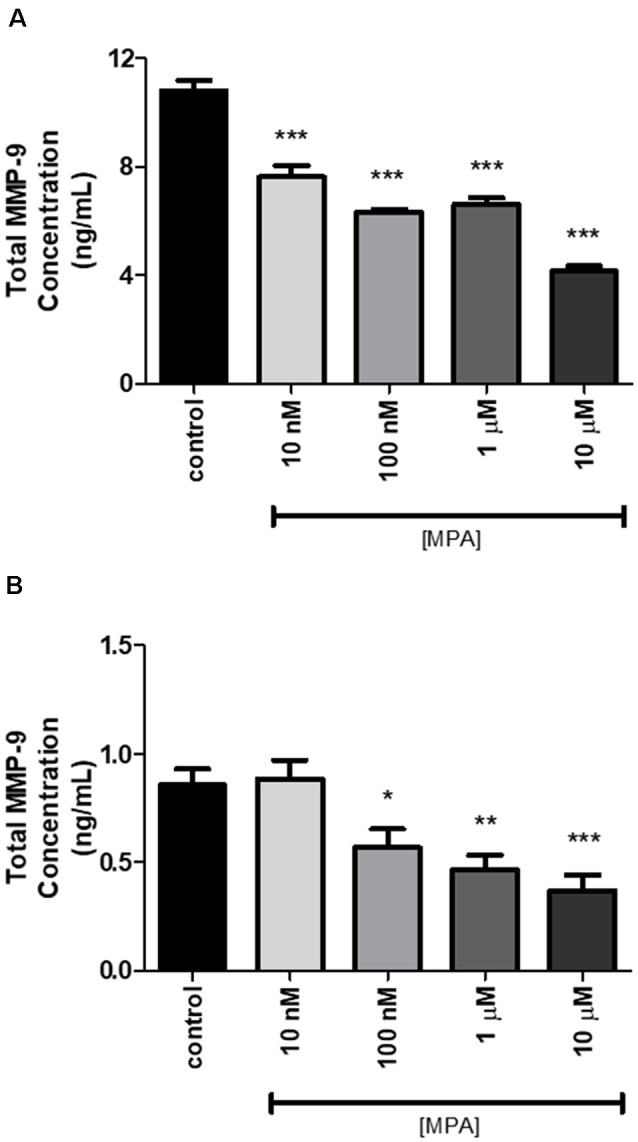
The effect of 48-h MPA treatment on MMP-9 protein in C6 glial cells. **(A)** Extracellular MMP-9 secretion was detected using the ELISA kit. MPA inhibited the expression of extracellular MMP-9 in treated C6 glial cells. Results are representative of three independent experiments. **(B)** The effect of 48-h MPA treatment on intracellular MMP-9 in C6 glial cells. MMP-9 secretion was detected using the ELISA kit. Results are expressed as means ± SEM. MPA inhibited the expression of intracellular MMP-9 in treated C6 glial cells. Results are representative of five independent experiments. *p < 0.05, **p < 0.01, and ***p < 0.001.

**Figure 3:**
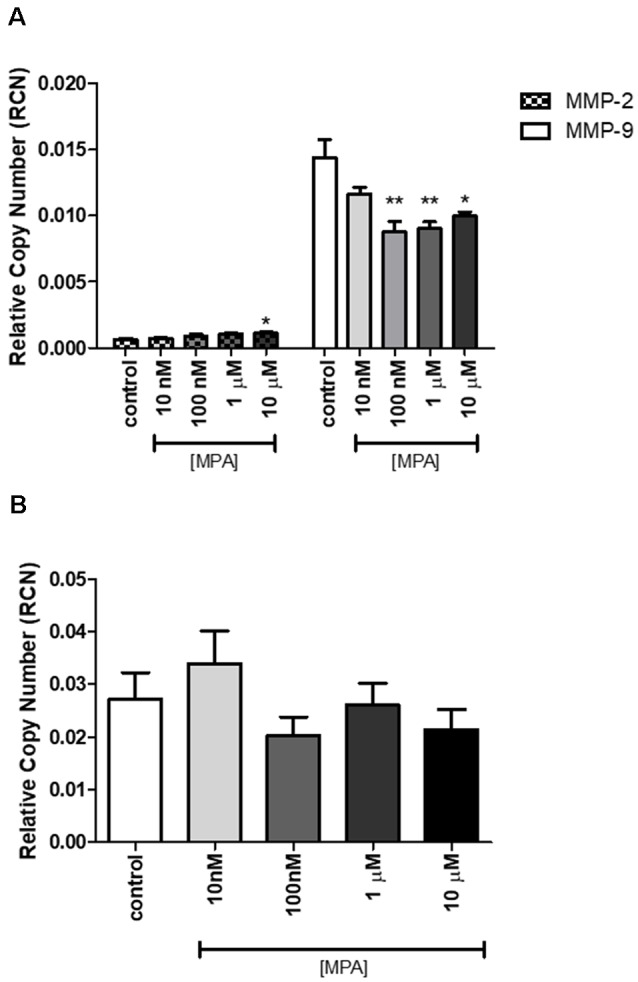
The effect of MPA treatment on MMP-2/-9 mRNA in C6 glial cells. **(A)** The mRNA levels of MMP-2 and MMP-9 were measured by qRT-PCR in C6 glial cells treated with MPA for 12-h, using MMP-2 and MMP-9 specific primers. **(B)** At 24-h, the suppression of MMP-9 mRNA levels is no longer detected. Data are represented by the mean ± SEM of three independent qRT-PCR experiments performed in duplicates. The expression levels are represented relative to the GAPDH reference gene. Results are representative of five independent experiments. *p < 0.05 and **p < 0.01.

Based on our finding that MPA caused decreased enzymatic activity of MMP-9, we next assessed whether the inhibitory effect of MPA on MMP-9 enzymatic activity was due to a reduction in the overall secretion of MMP-9 protein or TIMP-1 inhibition. To measure extracellular and intracellular MMP-9 protein levels, C6 cells were incubated for 48-h with increasing concentrations of the drug. Both the media and the cell lysates were collected and analyzed by ELISA for MMP-9 (pro-MMP-9, TIMP bound MMP-9, and latent MMP-9; Figures 2A,B). The analysis of three experimental replicates showed that MPA significantly inhibited extracellular MMP-9 protein levels (Figure 2A). MMP-9 inhibition ranged from 30% to 60% relative to control (Figure 2A). Intracellular MMP-9 protein expression was also significantly decreased after MPA treatment, in a dose-dependent manner (Control: 0.85548 ng/ml; 10 nM MPA: 0.88669 ng/ml; 100 nM MPA: 0.568602 ng/ml; 1 μM MPA: 0.468092 ng/ml; 10 μM MPA: 0.369965 ng/ml; Figure 2B). Because intracellular MMP-9 protein levels were negatively impacted by our MPA treatment, we evaluated the expression level of MMP-9 in cells treated with MPA by quantitative RT-PCR. Additionally, our zymography experiments revealed no detection of MMP-2 enzymatic activity, thus we performed an analysis of MMP-2 mRNA expression simply to confirm the lack of MMP-2 expression in the C6 cells (Figure 3A). Analysis of MMP-9 mRNA expression shows the experimental groups were significantly lower when compared with the control group (Figure 3A). Such repression appears after 12-h of treatment but is not detectable at later time points (24-h; Figure 3B).

### Medroxyprogesterone Acetate’s Effects Are Mediated Through the Glucocorticoid Receptor

**Figure 4:**
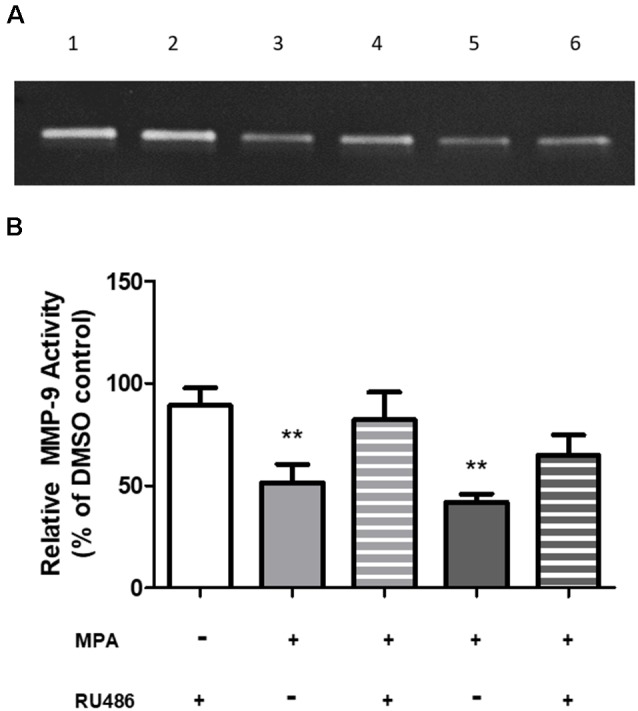
RU486 pretreatment antagonized the effects of MPA on MMP-9 activity. **(A)** Representative zymogram showing MMP-9 activity of MPA-treated conditioned media (CM). DMSO at 0.1% was used as the vehicle control and constitutively showed MMP-9 gelatinolytic activity (lane 1). C6 cells were pretreated with mifepristone, RU486 (2 μM), for 30 min, followed by MPA treatment for another 48-h. Treatment with RU486, alone, did not alter MMP-9 enzymatic activity (lane 2). Upon MPA treatment, MMP-9 gelatinolytic activity was significantly decreased (100 nM, lane 3; 10 μM, lane 5). Pretreatment with RU486 (2 μM) antagonized MPA’s effect on enzymatic activity (100 nM, lane 4; 10 μM, lane 6). **(B)** Densitometric analysis of conditioned media from astroglia determined by gelatin zymography. Results are expressed as percentage of activity of treated to untreated cells (mean ± SEM). MMP-9 gelatinolytic activity of untreated cells is expressed as 100%. RU486 treatment is represented by the white bar, 100 nM MPA treatments are represented by the light gray bars (solid and striped), 10 μM MPA treatments are represented by the dark gray bars (solid and striped). Results are representative of three independent experiments. **p < 0.01.

To investigate a potential mechanism by which MPA causes repression of MMP-9 transcription, we first considered a receptor-mediated approach. At the molecular level, MPA elicits its biological effects through multiple receptors, including the progesterone receptor (PR), androgen receptor (AR), and glucocorticoid receptor (GR; Africander et al., 2011). Notably, these cells are devoid of the PR and AR (Su et al., 2012b). The GR, however, is present in C6 cells and MPA binds to the GR with a high affinity, acting as a partial to a full agonist for the GR (Koubovec et al., 2004, 2005; Su et al., 2012a, b; Louw-du Toit et al., 2014). We tested the hypothesis that MPA’s effects on enzymatic activity and production of MMP-9 are mediated by the GR (Figure 4). C6 cells were pretreated with mifepristone, RU486 (2 μM), for 30 min, followed by MPA treatment for another 48-h, using the lowest and highest concentrations of MPA at which significant effects were seen on enzymatic activity, an intracellular protein, and mRNA levels (100 nM and 10 μM). Densitometric analysis of the zymogram (Figure 4A; Supplementary Figure S5) shows pharmacological inhibition of the GR with RU486 attenuated MPA’s effect on MMP-9 activity (100 nM MPA: 51.61%; 100 nM MPA/RU486: 82.23%; 10 μM: 41.99%; 10 μM MPA/RU486: 64.79% (Figure 4B). This suggests our findings are potentially due, in part, to a GR-mediated mechanism.

### Medroxyprogesterone Acetate Antagonizes the Degradation of Aβ

**Figure 5:**
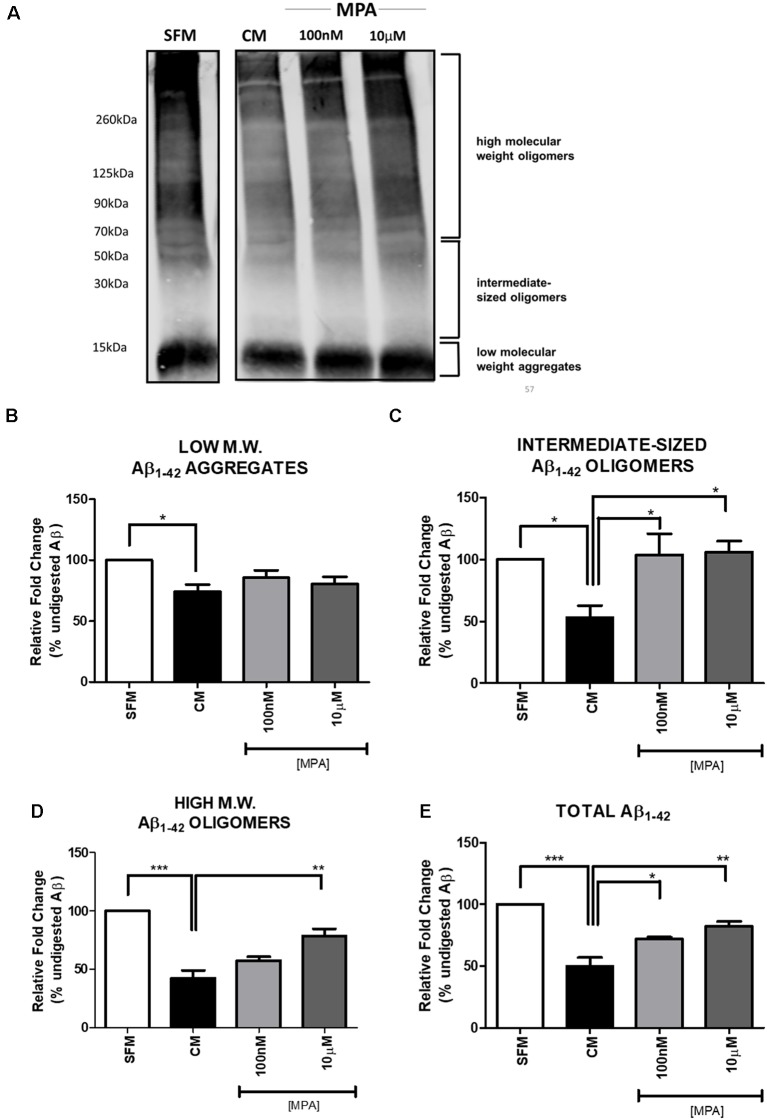
Western blot detection and densitometric analysis of Aβ using 6E10 antibody **(A)** Representative Western blot of Aβ using 6E10 antibody. Freshly prepared synthetic human Aβ_1–42_ (23 μM) was added to serum-free media (SFM; lane 1), SFM that had been conditioned by incubation with C6 glial cells (CM; lane 2), or CM that had been treated with MPA (100 nM or 10 μM) for 48-h (lanes 3 and 4). The mixture was then incubated for 24 h at 37°C, and residual Aβ was analyzed by Tris-Glycine—Western blotting. Incubation of Aβ_1–42_ with CM significantly decreased Aβ levels. MPA treatment attenuated this effect. **(B–E)** Densitometric analysis shows that MPA-treated CM samples induced significant differences in Aβ species. Incubation of Aβ_1–42_ with CM significantly decreased Aβ levels. MPA treatment attenuated this effect. Results are representative of three independent experiments. *p < 0.05, **p < 0.01, and ***p < 0.001.

Previous reports have suggested that MMP-9 is capable of degrading amyloid-beta *in vitro* (Backstrom et al., 1996). To explore the possibility that inhibition of MMP-9 would interfere with amyloid-beta degradation, C6 wells were treated with MPA for 48-h and the CM were incubated with freshly prepared synthetic human Aβ_1–42_ for 24-h at 37°C. The doses selected for this experiment were the lowest and the highest dose at which we observed significant effects on enzymatic activity, intracellular protein, and mRNA levels (100 nM and 10 μM). Using the anti-Aβ 6E10 antibody, Aβ levels were then measured by Tris-Glycine Gel-Western blotting (Figure 5A; Supplementary Figure S6). This approach yielded the resolution of groups of oligomers as indicated in Figure 5A, consistent with the literature (Prangkio et al., 2012). Incubation of Aβ with CM resulted in a significant reduction in Aβ levels, causing the appearance of several Aβ fragments, which are indicative of MMP cleavage (Backstrom et al., 1996). This Aβ-degrading activity was antagonized by MPA (100 nM and 10 μM). Densitometric analysis (Figures 5B–E) established that CM modestly altered low molecular weight aggregates (<15 kDa), while MPA-treated CM samples showed no effect (Figure 5B). However, MPA-treated CM samples overall mitigated proteolytic cleavage of intermediate-sized oligomers (~15–55 kDa; Figure 5C), high molecular weight oligomers (>56 kDa; Figure 5D), and total (Figure 5E) Aβ species. Incubation of Aβ_1–42_ with CM significantly abated Aβ levels and MPA treatment weakened this effect. To verify the contribution of MMPs to Aβ degradation in CM, we incubated CM with freshly prepared synthetic human Aβ_1–42_ for 24-h at 37°C in the presence or absence of the broad-spectrum, MMP inhibitor, GM6001, and found that the Aβ-degrading activity in CM was attenuated (Supplementary Figures S2, S7). Additionally, we incubated SFM with freshly prepare synthetic human Aβ_1–42_ for 24-h at 37°C in the presence or absence of active recombinant MMP-9 protein (rMMP-9). These data confirmed findings in the literature that MMP-9 possesses Aβ-degrading activity (data not shown; Backstrom et al., 1996; Yan et al., 2006; Hernandez-Guillamon et al., 2015). Next, we assessed the possibility of a direct action of MPA on Aβ. We treated SFM with MPA for 48-h. incubated the supernatant with 23 μM Aβ_1–42_, for an additional 24-h at 37°C and we found MPA-treated SFM failed to digest Aβ (data not shown). We determined our observed impairment of proteolytic degradation occurred by an indirect, cell-mediated mechanism.

## Discussion

Ninety percentage of the cells in the central nervous system (CNS) are glia (Haydon, 2001). Glial cells (astrocytes, oligodendrocytes, and microglia) communicate with neurons to regulate synaptic plasticity and neurotransmission (Fields et al., 2014). Accumulating evidence suggests MMP secretion from astroglia contributes to the degradation and clearance of amyloid plaques (Yan et al., 2006; Yin et al., 2006; Wang et al., 2014), emphasizing the critical role of induction and secretion of MMPs in the brain. There are several reports showing astroglia conditioned media (CM) possesses Aβ-degrading activity, in part, through the secretion of MMPs (Backstrom et al., 1996; Yin et al., 2006; Fragkouli et al., 2014). Moreover, MMP-9 possesses α-secretase-like activity and cleaves APP at several sites, promoting the non-amyloidogenic processing of the precursor protein and clearance of Aβ (Backstrom et al., 1996; Yin et al., 2006; Talamagas et al., 2007; Filippov and Dityatev, 2012).

The present study investigated the expression of MMP-9 in C6 glial cells treated with MPA. Several reports have demonstrated that MPA can alter MMP-9 levels, where levels are increased in macrophages and, alternatively, reduced in BV2 microglial, endometrial cancer, and primary amnion epithelial cells (Di Nezza et al., 2003; Hwang-Levine et al., 2011; Allen et al., 2019). Consistent with these reports, we found that MMP-9 protein expression and enzymatic activity is lowered by treatment with MPA. However, the majority of the previous *in vitro* studies have used non-CNS cell lines to observe the effect of MPA on MMP-9, with little to no investigation of the effects of MPA on glial secretion of MMP-9. Additionally, due to the lack of focus in a CNS-related system, previous work failed to investigate the potential for MPA to influence AD-related pathology via the hormonal modulation of MMP-9.

To our knowledge, this is the first *in vitro* analysis of the effect of MPA on MMP-9’s enzymatic activity and production, in a C6 glial cell line. Our results show that MMP-9 is expressed in C6 glial cells in control conditions and is suppressed by exposure to MPA at both the mRNA and the protein level. We found that MPA suppresses MMP-9 activity in a dose-dependent manner (Figure 1). Decreased MMP-9 activity was reflected by impaired synthesis of the MMP-9 protein (Figures 3A,B) and was further supported with data showing a suppression of MMP-9 mRNA (Figure 3A). We observed a spurious increase at the highest concentration of MPA (10 μM), which may be indicative of off-target effects because of such a high dose of the hormone. In agreement with our hypothesis, experiments on CM confirmed the inhibitory effects of MPA on the enzymatic activity of MMP-9. Conversely, we found MPA did not cause secretory inhibition of MMP-9, but rather suppressed the transcription of MMP-9 at the mRNA level.

The glucocorticoid receptor (GR) is typically found, in an inactive state, in the cytoplasm, and, upon ligand binding, it becomes and trans-represses pro-inflammatory genes. This is thought to be substantiated either through direct DNA-binding, by binding a glucocorticoid response element (GRE) or the nuclear factor kappa B (NF-κB) response element (NF κBRE; Meijsing et al., 2009; Surjit et al., 2011; Watson et al., 2013; Weikum et al., 2017; Hudson et al., 2018; Sacta et al., 2018) or 2), or through a DNA independent, direct protein-protein interaction/crosstalk with other transcription factors, including NF-κB and AP-1 (McEwan et al., 1997; Webster and Cidlowski, 1999; De Bosscher et al., 2003; Liu and Xu, 2012; Trevor and Deshane, 2014). The initial finding of MPA’s capability of interfering with the activities of NF-κB or AP-1, at the promoter level, suggested MPA represses cytokine-induced, AP-1 driven genes, as well as NF-κB-driven genes, without impacting the DNA-binding activity of NF-κB, in a GR-dependent manner (Koubovec et al., 2004). Conversely, Simoncini et al. (2004) demonstrated MPA’s anti-inflammatory effects and MPA’s ability to reduce hydrocortisone-dependent nuclear translocation of NF-κB in human endothelial cells. C6 cells have spontaneous NF-κB nuclear activity, suggesting the presence of constitutive NF-κB activity, which explains the constitutive basal expression of MMP-9 observed in our experiments (Robe et al., 2004). We treated C6 cells with a GR antagonist, which attenuated MPA’s effects on MMP-9 enzymatic activity (Figure 4). In this case, we suspect there may be a GR-dependent transrepression of MMP-9, *via* interaction with either NF-κB or AP-1, which are generally accepted as regulators of MMP-9 expression (Jonat et al., 1990; Paliogianni et al., 1993; Yokoo and Kitamura, 1996; Barnes, 1998; Bond et al., 2001; Ronacher et al., 2009; Africander et al., 2011; Li et al., 2012; Mittelstadt and Patel, 2012). The literature also extensively supports the notion that glucocorticoids alter MMP-9 expression (Rosenberg et al., 1996; Park et al., 1999; Eberhardt et al., 2002; de Paiva et al., 2006), as well as MMP-3 (Richardson and Dodge, 2003; Koyama et al., 2017), which is the enzyme responsible for the conversion of MMP-9 from an inactive to an active state. Therefore, it is plausible the progestin binds to the cytosolic GR, activates the GR, and then activated GR binds to NF-κB, in turn, negatively interfering with the transcriptional enhancer and, in turn, promoting transrepression of MMP-9. Notably, C6 cells have also been shown to express mRNA for the membrane progesterone receptors (mPR; Su et al., 2012a). Salazar et al. (2016) demonstrated MPA elicits progestin-induced intracellular signaling in PR-negative breast epithelial cells, suggesting a potential mode of action via membrane progesterone receptors. Possibly, our observations may be partially due to non-genomic signaling through membrane progesterone receptors, however, there is currently a lack of information regarding MPA’s relative binding affinity to membrane progesterone receptors and a lack of evidence relating to MPA’s propensity to elicit its effects via non-genomic membrane-bound signaling. Future studies should aim to further delineate the precise mechanisms by which our observations in the present study occur.

To our knowledge, this is the first investigation of the effects of MPA treatment on MMP-9’s Aβ-degrading activity. Our objective was to relate progestin-induced reductions of active MMP-9 levels with a loss of Aβ_1–42_ degradation. We were interested in observing the effects after exposure to high concentrations of Aβ (23 μM) and, using Western blot analysis, we were able to confirm the diminished ability of MPA-treated C6 cells to degrade Aβ_1–42_ (Figure 5). A key finding of our current study was that MPA-treated CM samples did not significantly alter low molecular weight aggregates (Figure 5B). However, MPA treatment impaired the degradation of intermediate-sized and high molecular weight oligomeric Aβ species. Of interest is the observed effect on oligomeric Aβ, which is suggested to be the culprit of the neurodegeneration seen in AD (Lesné et al., 2008, 2013; Lublin and Gandy, 2010). Our data suggest MPA treatment potentially promotes AD-related pathology. Our inclusion of the non-specific MMP inhibitor, GM6001, yielded a greater effect on degrading activity. This affirmed our observations were, in part, MMP-9 dependent, as GM6001 was shown to inhibit the enzymatic activity of MMP-9 by 80% (Figure 1). Enzymatic activity of MMP-9 was inhibited to the same extent by GM6001 and 10 μM MPA, which was reflected in their hindrance of Aβ degradation. Degradation of Aβ was not fully thwarted with GM6001 treatment, implicating the potential for additional proteases that exist in CM. As previously discussed, in addition to MMPs, Aβ can be degraded by other proteases, including ECE, IDE, and NEP (Saido and Leissring, 2012). Prior literature supports a minimal contribution of IDE, NEP, and ECE as extracellular, secreted amyloid-beta scavengers from astrocytes. According to Yin et al. (2006), in addition to NEP, ECE, and IDE, there may be proteases that have yet to be experimentally identified, contributing to astrocyte-mediated degradation of Aβ, because NEP, ECE, and IDE were undetected with Western blot analysis of conditioned media of neonatal mouse astrocytes. Moreover, it has been reported that cultured cells may be incapable of secreting IDE, and it is still undetermined whether NEP is capable of degrading oligomeric amyloid beta (Saido and Leissring, 2012; Song et al., 2018). There is evidence that estrogen promotes amyloid beta degradation through the induction of NEP (Liang et al., 2010), and there is also evidence that IDE is induced by 17β- estradiol, reducing amyloid beta load *in vivo* (Zhao et al., 2011). Thus, the literature generally supports the idea that hormones are capable of regulating these specific amyloid beta degrading enzymes and suggests MPA could also potentially regulate additional amyloid beta degrading enzymes. However, we focused on MMP-9, because MMP-9 is the only amyloid beta scavenger shown to possess the ability to degrade Aβ fibrils *in vitro*, Aβ plaques *in situ*, and Aβ *in vivo*, making it the most unique and distinguished of the known scavengers (Backstrom et al., 1996; Yan et al., 2006; Yin et al., 2006; Hernandez-Guillamon et al., 2015). Overall, our findings suggest MMPs are involved in the degradation of Aβ in CM and MPA impedes on the degradation, through the downregulation of MMP-9 production, in a GR-dependent manner.

We noted several limitations in the present study. First, our synthetic Aβ formulation and its aggregated forms may not fully represent the *in vivo* phenomena, due to variability in oligomer generation (refer to Supplementary Figure S7). Additionally, the effective concentrations of MPA in our study ranged from 10 nM to10 μM. Although our working concentrations are relatively high, peak serum concentrations of MPA fall between 10 nM and 100 nM (Tomasicchio et al., 2013), after women receive an intramuscular injection of 150 mg, every 3 months. These are concentrations at which our observed effects on MMP-9 and Aβ-degrading activity occurred. Furthermore, our i*n vitro* model utilizes a transformed glial cell line. The utilization of primary cells are more reliable than cell lines, however, this would require pharmacological induction of MMP-9 expression or transfection of an MMP-9 expression vector. As previously mentioned, C6 cells have spontaneous NF-κB nuclear activity, suggesting the presence of constitutive NF-κB activity, which explains the constitutive basal expression of MMP-9 observed in our experiments (Robe et al., 2004), and circumvented the necessity for induction or transfection of MMP-9 expression. Although *in vitro* experiments using cell culture are useful, providing pertinent information, ultimately *in vivo* experiments are needed to confirm these effects.

Based on our findings, progestin-induced downregulation of MMPs is partially responsible for hampering the proteolytic cleavage of Aβ_1–42_ and supports a possible link between MPA administration and AD-related pathology. The importance of MMP-mediated degradation of toxic Aβ_1–42_ species and its potential neuroprotective effect is abolished with MPA-induced reduction in MMP-9 expression and production. Most importantly, this commonly used progestin suppresses both the transcription and the activation of glial MMP-9, which is responsible for reduced Aβ degradation. Taken together, our study confirmed Aβ-degrading activity was reduced, in part, through the impairment of MMP-9 production.

In summary, our work demonstrates the necessity for a further delineation of MPA’s effects on MMP-9 production. We also raise awareness for the unmet need for *in vivo* investigation for the potential cognitive and pathological outcomes of MPA. There is currently an abundance of literature which focuses on the effects of estrogen, estradiol, testosterone, and progesterone on the brain, and particularly amyloid-beta production and clearance (Vest and Pike, 2013; Li and Singh, 2014; Giatti et al., 2016; Uchoa et al., 2016). There is still a paucity of published research addressing the potential effects of progestins, more specifically MPA, on the brain. While limited research with MPA suggests a negative impact on the brain, these studies need to be extended to model systems relevant to women in their reproductive prime, as a majority of efforts have been in menopausal animal models and menopausal woman (Shumaker et al., 2003, 2004; Braden et al., 2010, 2011, 2017; Lowry et al., 2010; Akinloye Olanrewaju et al., 2013). Our finding that MPA limits MMP-9 production could ultimately negatively impact synaptic plasticity *in vivo*, as MMP-9 is considered “indispensable” for neuronal plasticity (Nagy et al., 2006; Michaluk et al., 2011; Kamat et al., 2014; Gorkiewicz et al., 2015; Lepeta and Kaczmarek, 2015; Kaczmarek, 2016). Thus, independent of AD-related pathology, MPA’s suppression of MMP-9 production could still prove harmful for learning and memory. Herein, we encourage additional investigations related to MPA’s effects on the brain. Future efforts should be extended to model systems relevant to AD-related pathology. It is conceivable that the prolonged use of MPA will progressively subdue the proteolytic degradation of Aβ by MMP-9, *in vivo*, promoting AD-related pathology. This hypothesis is currently being tested in our laboratories.

## Data Availability Statement

The datasets generated for this study are available on request to the corresponding author.

## Author Contributions

KP designed studies, conducted studies, and composed the manuscript. SS, DD, MV, and DQ aided with studies and revised the manuscript. JS designed studies and revised the manuscript.

## Conflict of Interest

The authors declare that the research was conducted in the absence of any commercial or financial relationships that could be construed as a potential conflict of interest.

## Supplementary Material

FIGURE S1 Effect of MPA on cell viability and LDH release. **(A)** Calcein AM and **(B)** Lactate dehydrogenase (LDH) data indicate that significant cell death did not occur at any concentration of MPA treatment.

FIGURE S2 Western blot detection and densitometric analysis of Aβ using 6E10 antibody. Representative Western blot of Aβ using 6E10 antibody. Freshly prepared synthetic human Aβ_1–42_ was added to serum-free media (SFM; lane 1), SFM that had been conditioned by incubation with C6 glial cells (CM; lane 2), or CM that had been treated with 1 μM GM6001 (lane 3) for 48-h. The mixture was then incubated for 24 h at 37°C, and residual Aβ was analyzed by **(A)**, Tris-Glycine—Western blotting. **(B)** Densitometric analysis of overall total Aβ_1–42_shows that incubation of Aβ_1–42_ with CM significantly decreased Aβ levels and GM6001 treatment attenuated this effect. Results are representative of two independent experiments. **p* < 0.05, ***p* < 0.01.

FIGURE S3 Full Zymogram gel of cropped image as shown in Figure 1A. Using a 10-well gel, MMP-9 Active, Human, Recombinant (Millipore Sigma; #PF024-5UG) was used as a reference standard showed MMP-9 gelatinolytic activity at 67 kDa (lane 1). DMSO at 0.1% was used as the vehicle control and constitutively showed MMP-9 gelatinolytic activity (lane 3). Upon MPA treatment, MMP-9 gelatinolytic activity was significantly decreased (lanes 4–7). GM6001 (lane 8) was used as a negative control for active MMP-9.

FIGURE S4 Full Zymogram gel of cropped image as shown in Figure 1B. Using a 12-well gel, DMSO at 0.1% was used as the vehicle control and constitutively showed MMP-9 gelatinolytic activity (lanes 1 and 7). Upon MPA treatment, MMP-9 gelatinolytic activity was significantly decreased (lanes 2–5 and 8–11). GM6001 (lanes 6 and 12) was used as a negative control for active MMP-9.

FIGURE S5 Full Zymogram gel of cropped image as shown in Figure 4. Using a 10-well gel, DMSO at 0.1% was used as the vehicle control and constitutively showed MMP-9 gelatinolytic activity (lane 1). C6 cells were pretreated with mifepristone, RU486 (2 μM), for 30 min, followed by MPA treatment for another 48-h. Treatment with RU486, alone, did not alter MMP-9 enzymatic activity (lane 2). Upon MPA treatment, MMP-9 gelatinolytic activity was significantly decreased (100 nM, lane 3; 10 μM, lane 5). Pre-treatment with RU486 (2 μM) antagonized MPA’s effect on enzymatic activity (100 nM, lane 4; 10 μM, lane 6).

FIGURE S6 Full Western Blot gel of cropped image as shown in Figure 5A. Li-Cor Pre-Stained Protein Ladder (Chameleon^™^ Duo; #928-60000; lane 1). Freshly prepared synthetic human Aβ1–42 (23 μM) was added to serum-free media (SFM; lane 3), 10% fetal bovine serum (FBS) media (lane 4), SFM that had been conditioned by incubation with C6 glial cells (CM; lane 5), or to 100 nM MPA-treated CM (lane 6) or 10 μM MPA-treated CM (lane 7).

FIGURE S7 Full Western Blot gel of cropped image as shown in Supplementary Figure S2. Li-Cor Pre-Stained Protein Ladder (Chameleon^™^ Duo; #928-60000; lane 1). Freshly prepared synthetic human Aβ^1–42^ (23 μM) was added to serum-free media (SFM; lane 2), SFM that had been conditioned by incubation with C6 glial cells (CM; lane 3), CM that had been treated with 1 μM GM6001 (lane 5), or to CM that had been treated with MPA (10 nM, 100 nM, 1 μM, and 10 μM) for 48-h (lanes 7–10). As noted in our “Discussion”, our synthetic Aβ preparation exhibited variability in oligomer generation. The variability in aggregates stems from using HPLC grade water as our solvent, instead of alcohol, such as hexafluoroisopropanol (HFIP), which removes preexisting aggregates and beta-sheet secondary structures from Aβ^1–42^, yielding the peptide in one specific form of Aβ^1–42^ species. The solvent used to dissolve the lyophilized peptides determines the initial conformation of amyloid-beta and also the aggregation kinetics (Wei and Shea, 2006). Additionally, while performing replicates of our studies, our amyloid preparation was stored at −80. Amyloid peptides are shown to be sensitive to temperature and freezing even at low concentrations, where freezing the peptide allows for uncontrolled oligomerization, causing the variation in aggregates once it goes through a freeze/thaw cycle (Filippov et al., 2008). We observed changes in the preparation as an effect of time and temperature. During our experiments and replicates, we found that if the preparation was not freshly made with each replicate, our Western blot detection would then lack the sensitivity required to detect MPA-induced effects on degradation. The effects of CM and GM600, however, could still be detected, using a synthetic preparation that was not freshly made.
