# Supplementary figures and images for: Medroxyprogesterone Acetate Impairs Amyloid Beta Degradation in a Matrix Metalloproteinase-9 Dependent Manner
Source: Front Aging Neurosci. 2020 Apr 7;12:92. doi: 10.3389/fnagi.2020.00092 (PMC7155169; doi:10.3389/fnagi.2020.00092)

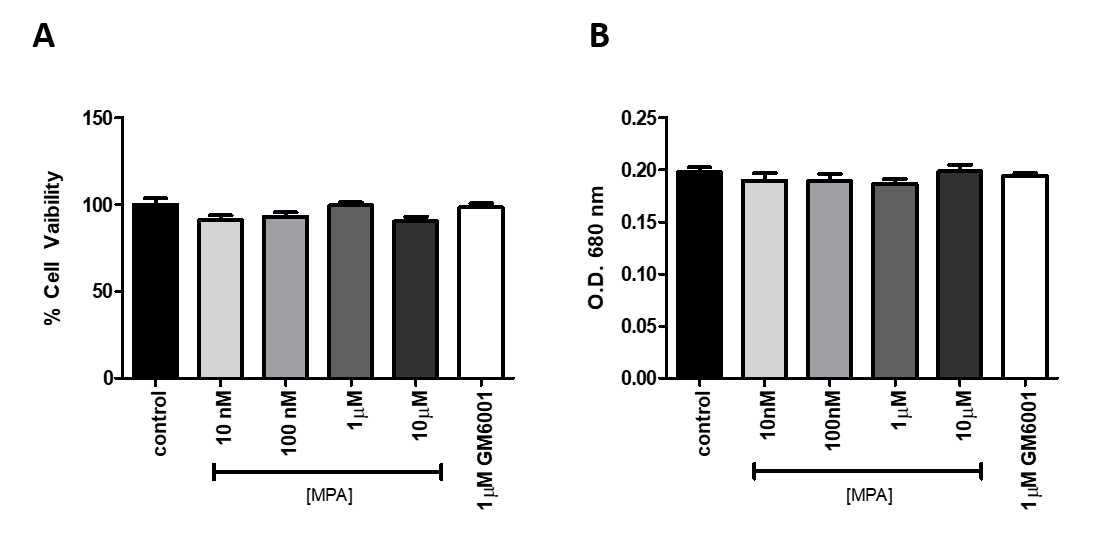

Supplement: FIGURE S1 — Effect of MPA on cell viability and LDH release. (A) Calcein AM and (B) Lactate dehydrogenase (LDH) data indicate that significant cell death did not occur at any concentration of MPA treatment. [file Image_1.TIF]

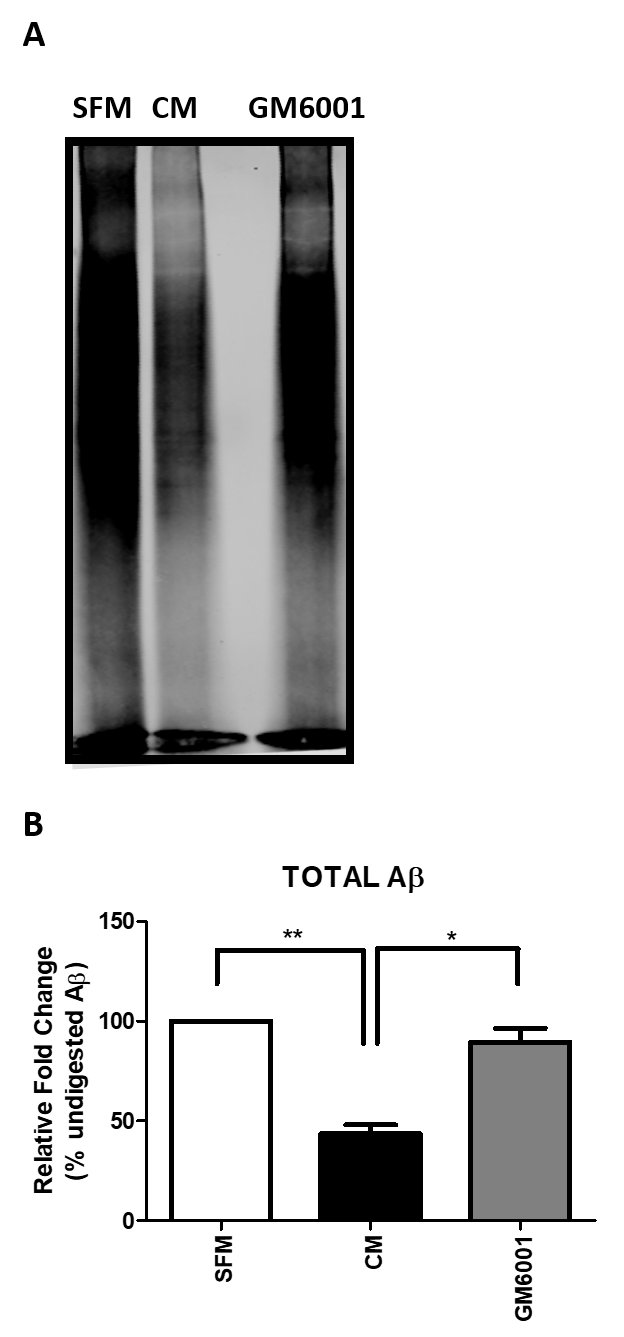

Supplement: FIGURE S2 — Western blot detection and densitometric analysis of Aβ using 6E10 antibody. Representative Western blot of Aβ using 6E10 antibody. Freshly prepared synthetic human Aβ1–42 was added to serum-free media (SFM; lane 1), SFM that had been conditioned by incubation with C6 glial cells (CM; lane 2), or CM that had been treated with 1 μM GM6001 (lane 3) for 48-h. The mixture was then incubated for 24 h at 37°C, and residual Aβ was analyzed by (A), Tris-Glycine—Western blotting. (B) Densitometric analysis of overall total Aβ1–42shows that incubation of Aβ1–42 with CM significantly decreased Aβ levels and GM6001 treatment attenuated this effect. Results are representative of two independent experiments. *p < 0.05, **p < 0.01. [file Image_2.TIF]

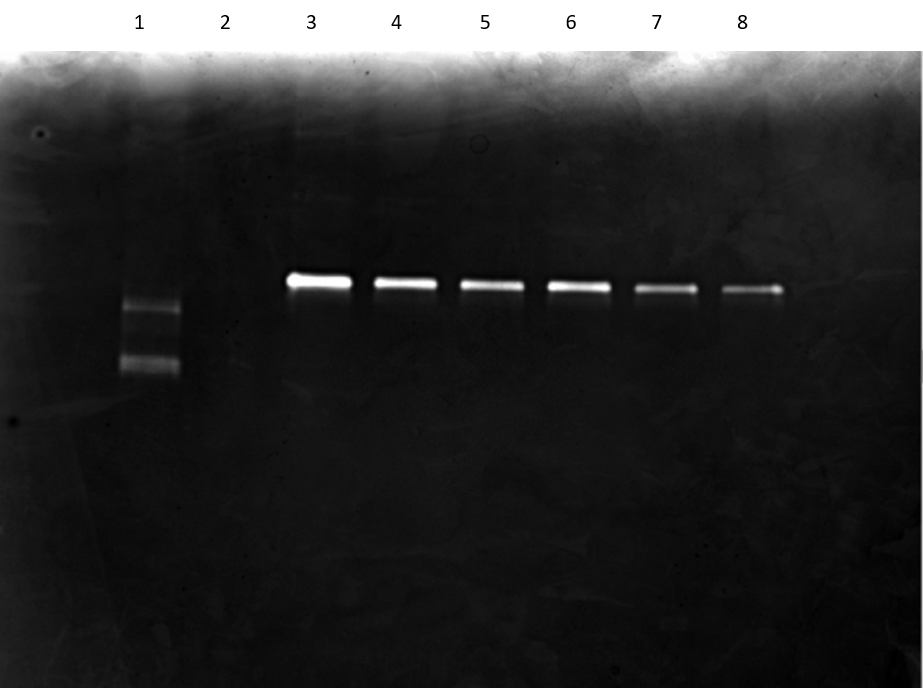

Supplement: FIGURE S3 — Full Zymogram gel of cropped image as shown in Figure 1A. Using a 10-well gel, MMP-9 Active, Human, Recombinant (Millipore Sigma; #PF024-5UG) was used as a reference standard showed MMP-9 gelatinolytic activity at 67 kDa (lane 1). DMSO at 0.1% was used as the vehicle control and constitutively showed MMP-9 gelatinolytic activity (lane 3). Upon MPA treatment, MMP-9 gelatinolytic activity was significantly decreased (lanes 4–7). GM6001 (lane 8) was used as a negative control for active MMP-9. [file Image_3.TIF]

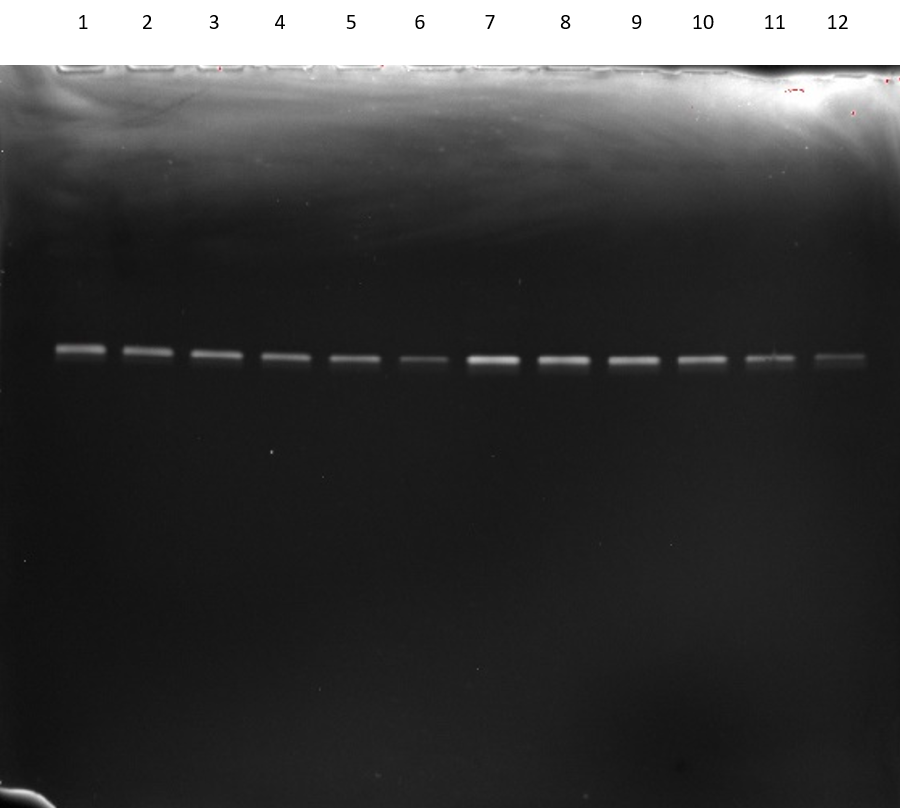

Supplement: FIGURE S4 — Full Zymogram gel of cropped image as shown in Figure 1B. Using a 12-well gel, DMSO at 0.1% was used as the vehicle control and constitutively showed MMP-9 gelatinolytic activity (lanes 1 and 7). Upon MPA treatment, MMP-9 gelatinolytic activity was significantly decreased (lanes 2–5 and 8–11). GM6001 (lanes 6 and 12) was used as a negative control for active MMP-9. [file Image_4.TIF]

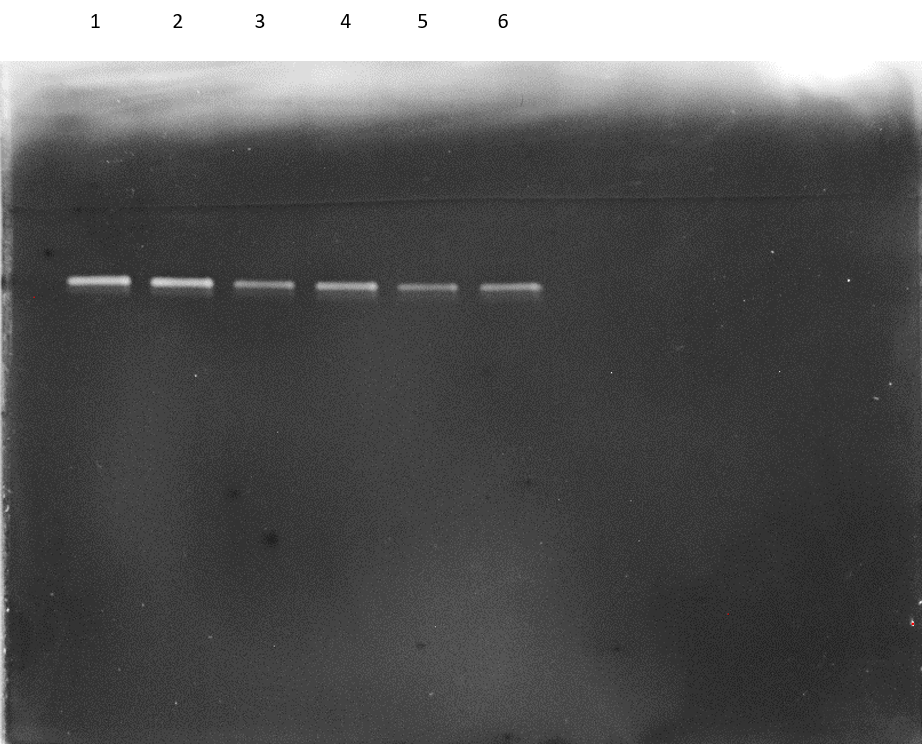

Supplement: FIGURE S5 — Full Zymogram gel of cropped image as shown in Figure 4. Using a 10-well gel, DMSO at 0.1% was used as the vehicle control and constitutively showed MMP-9 gelatinolytic activity (lane 1). C6 cells were pretreated with mifepristone, RU486 (2 μM), for 30 min, followed by MPA treatment for another 48-h. Treatment with RU486, alone, did not alter MMP-9 enzymatic activity (lane 2). Upon MPA treatment, MMP-9 gelatinolytic activity was significantly decreased (100 nM, lane 3; 10 μM, lane 5). Pre-treatment with RU486 (2 μM) antagonized MPA’s effect on enzymatic activity (100 nM, lane 4; 10 μM, lane 6). [file Image_5.TIF]

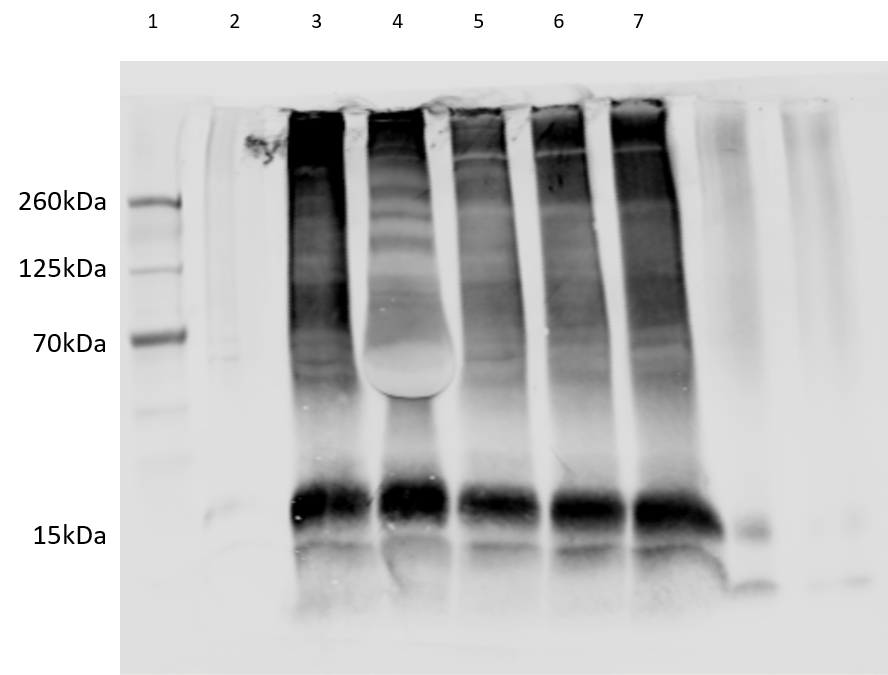

Supplement: FIGURE S6 — Full Western Blot gel of cropped image as shown in Figure 5A. Li-Cor Pre-Stained Protein Ladder (Chameleon™ Duo; #928-60000; lane 1). Freshly prepared synthetic human Aβ1–42 (23 μM) was added to serum-free media (SFM; lane 3), 10% fetal bovine serum (FBS) media (lane 4), SFM that had been conditioned by incubation with C6 glial cells (CM; lane 5), or to 100 nM MPA-treated CM (lane 6) or 10 μM MPA-treated CM (lane 7). [file Image_6.TIF]

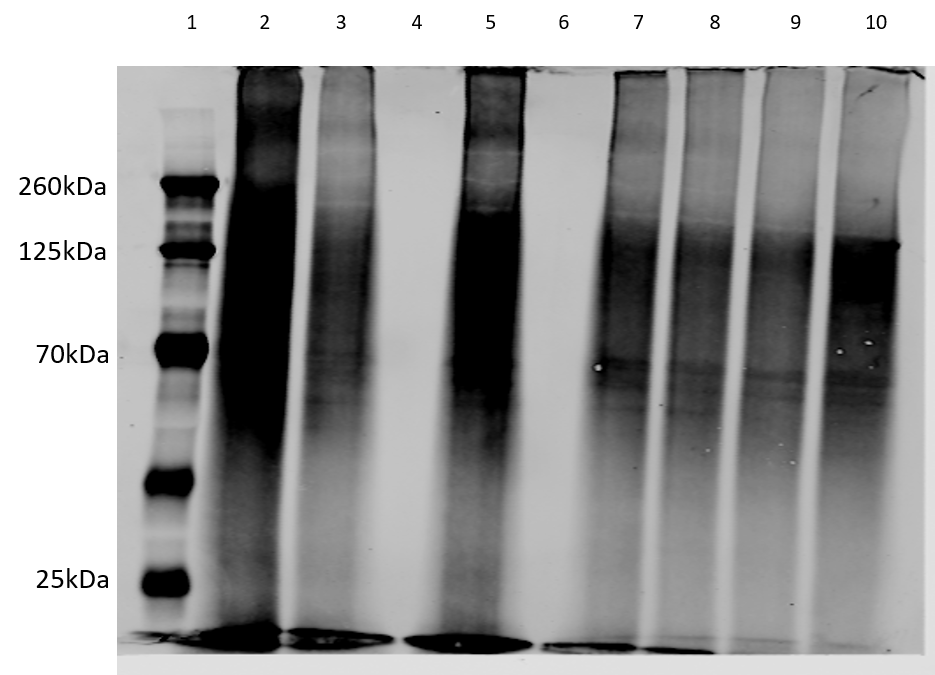

Supplement: FIGURE S7 — Full Western Blot gel of cropped image as shown in Supplementary Figure S2. Li-Cor Pre-Stained Protein Ladder (Chameleon™ Duo; #928-60000; lane 1). Freshly prepared synthetic human Aβ1–42 (23 μM) was added to serum-free media (SFM; lane 2), SFM that had been conditioned by incubation with C6 glial cells (CM; lane 3), CM that had been treated with 1 μM GM6001 (lane 5), or to CM that had been treated with MPA (10 nM, 100 nM, 1 μM, and 10 μM) for 48-h (lanes 7–10). As noted in our “Discussion”, our synthetic Aβ preparation exhibited variability in oligomer generation. The variability in aggregates stems from using HPLC grade water as our solvent, instead of alcohol, such as hexafluoroisopropanol (HFIP), which removes preexisting aggregates and beta-sheet secondary structures from Aβ1–42, yielding the peptide in one specific form of Aβ1–42 species. The solvent used to dissolve the lyophilized peptides determines the initial conformation of amyloid-beta and also the aggregation kinetics (Wei and Shea, 2006). Additionally, while performing replicates of our studies, our amyloid preparation was stored at −80. Amyloid peptides are shown to be sensitive to temperature and freezing even at low concentrations, where freezing the peptide allows for uncontrolled oligomerization, causing the variation in aggregates once it goes through a freeze/thaw cycle (Filippov et al., 2008). We observed changes in the preparation as an effect of time and temperature. During our experiments and replicates, we found that if the preparation was not freshly made with each replicate, our Western blot detection would then lack the sensitivity required to detect MPA-induced effects on degradation. The effects of CM and GM600, however, could still be detected, using a synthetic preparation that was not freshly made. [file Image_7.TIF]
